# The malignant stroke indicator is an early indicator of malignant ischemic stroke requiring decompressive hemicraniectomy

**DOI:** 10.1038/s41598-025-92284-2

**Published:** 2025-03-04

**Authors:** Xenia Hautmann, Ala Jamous, Ilko Maier, Christoph Bettag, Silvia Hernandez Duran, Ruslan Muradzade, Dorothee Mielke, Veit Rohde, Vesna Malinova

**Affiliations:** 1 Department of Neurosurgery, University Medical Center Göttingen, Robert-Koch Straße 40, 37075 Göttingen, Germany; 2 Department of Neuroradiology, University Medical Center Göttingen, Göttingen, Germany; 3 Department of Neurology, University Medical Center Göttingen, Göttingen, Germany; 4 Department of Neurosurgery, University Hospital Augsburg, Augsburg, Germany

**Keywords:** Malignant ischemic stroke, Decompressive hemicraniectomy, Brain edema, Stroke, Risk factors

## Abstract

Decompressive craniectomy (DHC) can prevent mortality in patients with malignant ischemic stroke. However, no clear criteria have been established to early identify patients, who will develop malignant stroke requiring DHC. In this retrospective observational study, a large patient cohort with ischemic stroke treated between 2010 and 2021, was analyzed. Clinical and radiological parameters were analyzed. Univariate and multivariate regression analyses were performed to identify the parameters to be included in the score. A cohort of 534 patients was included. A malignant stroke indicator (MSI) score was created including age < 70 years with 7 points, midline shift with up to 6 points, unsuccessful recanalization (TICI < 2b) with 6 points, basal cistern effacement with 4 points, and CBV ASPECTS < 6 with 3 points assigned. A MSI score with a cutoff value of 9 showed a high discrimination power concerning the need for DHC (AUC 0.90, *p* < 0.0001). Patients with MSI-score ≥ 9 had a 22-fold higher probability of needing DHC (odds ratio 22.90, *p* < 0.0001). The MSI score is a promising tool to predict the need for DHC in patients at risk for developing a malignant stroke and needs to be validated in external cohorts.

## Introduction

Ischemic stroke counts to the leading causes of death and disability worldwide. Up to 10% of patients with cerebral infarction of the anterior circulation develop malignant edema leading to cerebral herniation and resulting into mortality rates of up to 80% during the first week after ictus^1,2^. Decompressive hemicraniectomy (DHC) significantly increases one-year survival from 29 to 78% compared to conservative treatment alone^3,4^. However, the morbidity of the surviving patients remains high. Good functional outcome with mild or moderate disability was previously reported in only 10% and 33% of patients receiving DHC, respectively^5^. A lack of reliable parameters allowing an early recognition of stroke patients at high risk for developing malignant edema with the consequence of not timely performed DHC could be at least partly the explanation for these findings. The results of three randomized-controlled trials – DESTINY^6^, HAMLET^7^ and DECIMAL^8^ – have provided evidence, that DHC should be performed within 48 h after ictus in younger patients (< 60 years old). A few scores for prediction of malignant edema in patients with ischemic stroke have been published so far^9,10^. The Enhanced Detection of Edema in Malignant Anterior Circulation Stroke (EDEMA)-score was developed by Ong et al. to predict potentially lethal malignant edema in patients with an infarction of the anterior circulation^9^. The Alberta Stroke Program Early CT (ASPECT)-score helps in estimating the size of ischemic brain tissue, which is usually considered for the indication of thrombectomy^10,11^. Both scores showed a discrimination power of 0.76 for brain edema, but they haven’t been evaluated regarding the indication of DHC yet. A reliable risk stratification tool allowing an early recognition of patients at high risk for malignant edema requiring DHC still needs to be defined. The purpose of this study was to assess the discrimination power of clinical and radiological parameters indicating a high risk for malignant ischemic stroke with the aim of developing a score specifically for an early identification of stroke patients needing DHC.

## Methods

### Study population and study design

In this retrospective observational study, a consecutive patient cohort with ischemic stroke due to an occlusion of the middle cerebral artery or the internal carotid artery, treated at our center between 2010 and 2021 was analyzed. Only adult patients (≥ 18 years) with a National Institutes of Health Stroke Scale (NHISS) > 7 at baseline with symptom onset within the last 24 h, and a computed tomography (CT)-scan performed on admission, were eligible for inclusion in the study. Patients were excluded if stroke treatment (intravenous thrombolysis, thrombectomy or DHC) was primarily declined according to personal directive of the patients to avoid a misinterpretation of the results. Further exclusion criteria were age younger than 18, NIHSS ≤ 7, intracerebral hemorrhage, ischemic stroke of the posterior circulation or incomplete data (e.g., unclear onset of stroke). Modified Rankin scale (mRS) at 3-months follow-up was a secondary outcome parameter in this study.

The study was approved by the local ethics committee of the University Medical Center, Göttingen (Number 6/10/23). Due to the retrospective nature of the study, the local ehtics commitee of the University Medical Center Göttingen waived the need of obtaining informed consent.

### Treatment protocol for patients with ischemic stroke at our center

Stroke management in our center is performed in line with the current guidelines for acute stroke. If an LVO is detected, emergency mechanical thrombectomy is performed after case discussion between the consultant neurologist and neuro-interventionalist. If the onset of symptoms is less than 4.5 h, intravenous administration of recombinant tissue plasminogen activator (rt-PA) is given. After MT the patient is treated according to current neurological guidelines on a certified stroke unit or neurointensive care unit. A follow-up CT scan is routinely performed 24 h after MT or whenever a neurological deterioration occurs. In case of progressive brain edema with midline-shift and/or decrease of consciousness, the attending neurosurgeon is informed. The final decision whether to perform a DHC or not was left to the discretion of the neurosurgeon on call based on an interdisciplinary case-to-case assessment.

### Identification of reliable malignant stroke indicators

Several parameters, that were previously reported in studies to be associated with malignant ischemic stroke possibly requiring DHC, were included in the analysis of the study: age^11^, previous stroke^11^, basal cistern effacement^9^, midline shift^12^, successful recanalization^9^, i.e., MT defined as Thrombolysis In Cerebral infarction (TICI)-scale ≥ 2b^9^, ischemic signs on native computed tomography (NCCT ASPECTS)^13^ as well as on CT perfusion (CBV ASPECTS)^13^, clinical status according to NIHSS at baseline^11^, and the collateral circulation according to the grading defined by Souza et al.^12^.

### Elaboration of a scoring system

The primary outcome parameter was the need for DHC. All significant parameters in the univariate regression analysis were included into a multivariate regression model to identify independent predicting factors of malignant stroke requiring DHC. A malignant stroke indicator (MSI) score was elaborated based on the identified independent predictors assigning points according to the discrimination power of each included parameter. The discriminatory power of the MSI score was evaluated in the total population as well in subgroup analyses separating the patients with successful thrombectomy, which was defines as TICI ≥ 2b from those without successful thrombectomy defined as TICI < 2 b.

### Correlation of the MSI score with functional outcome at 3 months follow up

Additionally, the correlation of the MSI scoring system with functional outcome at 3 months follow up was examined and its ability to discriminate patients with favorable functional outcome at 3 months follow up defined as mRS ≤ 3 from those with unfavorable outcome was assessed.

### Statistical analysis

The statistical analyses were performed by means of the GraphPad Prism software (Version 10, GraphPad Software, San Diego, CA, USA). For the presentation of baseline data descriptive statistics and frequency distribution analysis was done. Continuous variables are depicted as mean ± standard deviation (SD), categorical variables as frequency or percentages. Descriptive statistics was used for calculation of baseline characteristics in the study population. A p-value of < 0.05 was considered significant. Univariate regression analysis was performed to identify parameters associated with the need for DHC. All significant parameters from the univariate analysis were than included into a multivariate regression model to identify independent predictors. Receiver operating curve (ROC) – analysis was done to determine the cutoff values separating the patients with malignant stroke needing DHC from those who don’t. Area under the curve (AUC), sensitivity, specificity, positive predictive value, and negative predictive value were calculated to evaluate the discriminatory power of the scoring system. The same procedure was then followed for the subgroups with and without successful thrombectomy. We correlated our Score for each patient with the mRS using a two-tailed Spearman´s correlation and performed ROC analysis to determine cutoff values separating patients with favorable and unfavorable outcome.

### Previous presentation

Some of these results were presented at the section meeting of the German Society for Neurosurgery in Weimar in October 2023.

## Results

### Patient population

A total of 534 patients with ischemic stroke due to an occlusion of middle cerebral artery (356/534; 67%) or internal carotid artery (178/534; 33%) were included in the study. The mean age was 71.05 ± 13.69 years (range 23–101). Mean NIHSS at baseline was 15.64 ± 6.08. DHC was performed in 29% (153/534) of all included patients. A successful recanalization was achieved in 68% (361/534) of all patients. Good functional outcome with mild disability (mRS ≤ 2) was found in 26% of all patients. Mean mRS in the study population was 3.5 ± 1.7 (median 4, 95%CI 3–4). Baseline characteristics of the study population are summarized in Table 1.

**Table 1:** Baseline characteristics.

Parameters	All	No DHC - group	DHC - group	*p*-value
Number of patients	534	381	153	
Mean age ± SD	71.05 ± 13.69	75.23 ± 12.08	60.64 ± 11.83	< 0.0001*
Sex % (n)				0.01*
Male	51% (272/534)	48% (181/381)	59% (91/153)	
Female	49% (262/534)	52% (200/381)	41% (62/153)	
NIHSS on admission Mean ± SD	15.64 ± 6.08	14.99 ± 6.23	17.26 ± 5.36	< 0.0001*
rt-PA lysis % (n)				0.30
No	46% (246/534)	45% (170/381)	50% (76/153)	
Yes	54% (287/534)	55% (210/381)	50% (77/153)	
Successful recanalization % (n) (TICI ≥ 2b)				< 0.0001*
No	32% (173/534)	18% (67/381)	69% (106/153)	
Yes	68% (361/534)	82% (314/381)	31% (47/153)	
NCCT ASPECTS Mean ± SD	7.14 ± 2.92	8.02 ± 1.03	4.94 ± 3.59	< 0.0001*
CBV ASPECTS Mean ± SD	6.21 ± 2.92	6.76 ± 2.67	4.33 ± 2.97	< 0.0001*

TICI = thrombolysis in cerebral infarction, NCCT = native cranial computed tomography, CBV = cerebral blood volume, ASPECTS = Alberta stroke program early CT score, NIHSS = National Institutes of Health Stroke Scale.

*Significance level is *p* < 0.05.

### Malignant stroke indicator score for identification of stroke patients requiring DHC

**Fig. 1:**
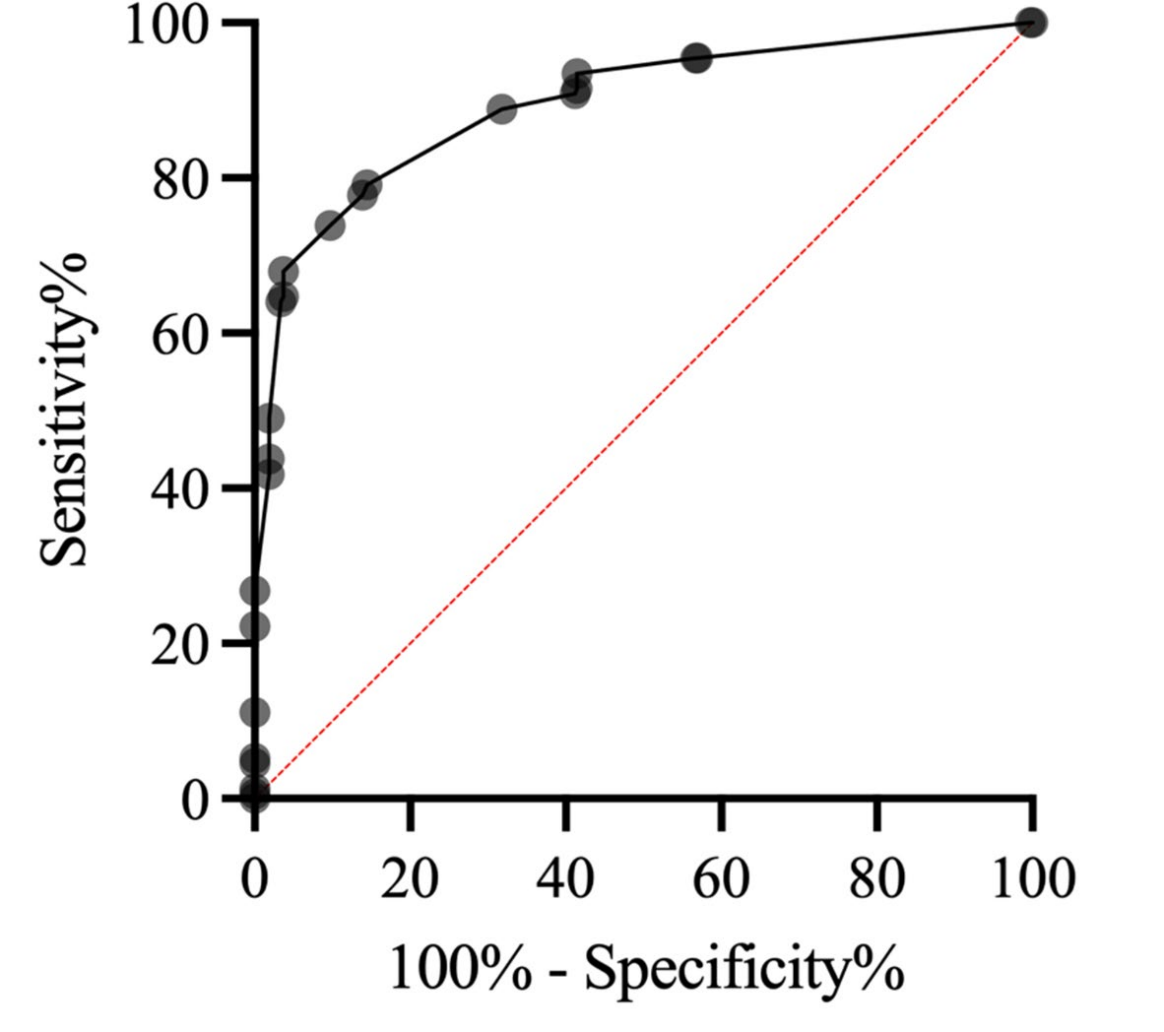
ROC-analysis of the MSI-score for the discrimination of patients requiring DHC and those, who don’t (AUC 0.90, 95%CI 0.87–0.93; *p* < 0.0001) with a cutoff value of ≤ 9 points.

**Fig. 2:**
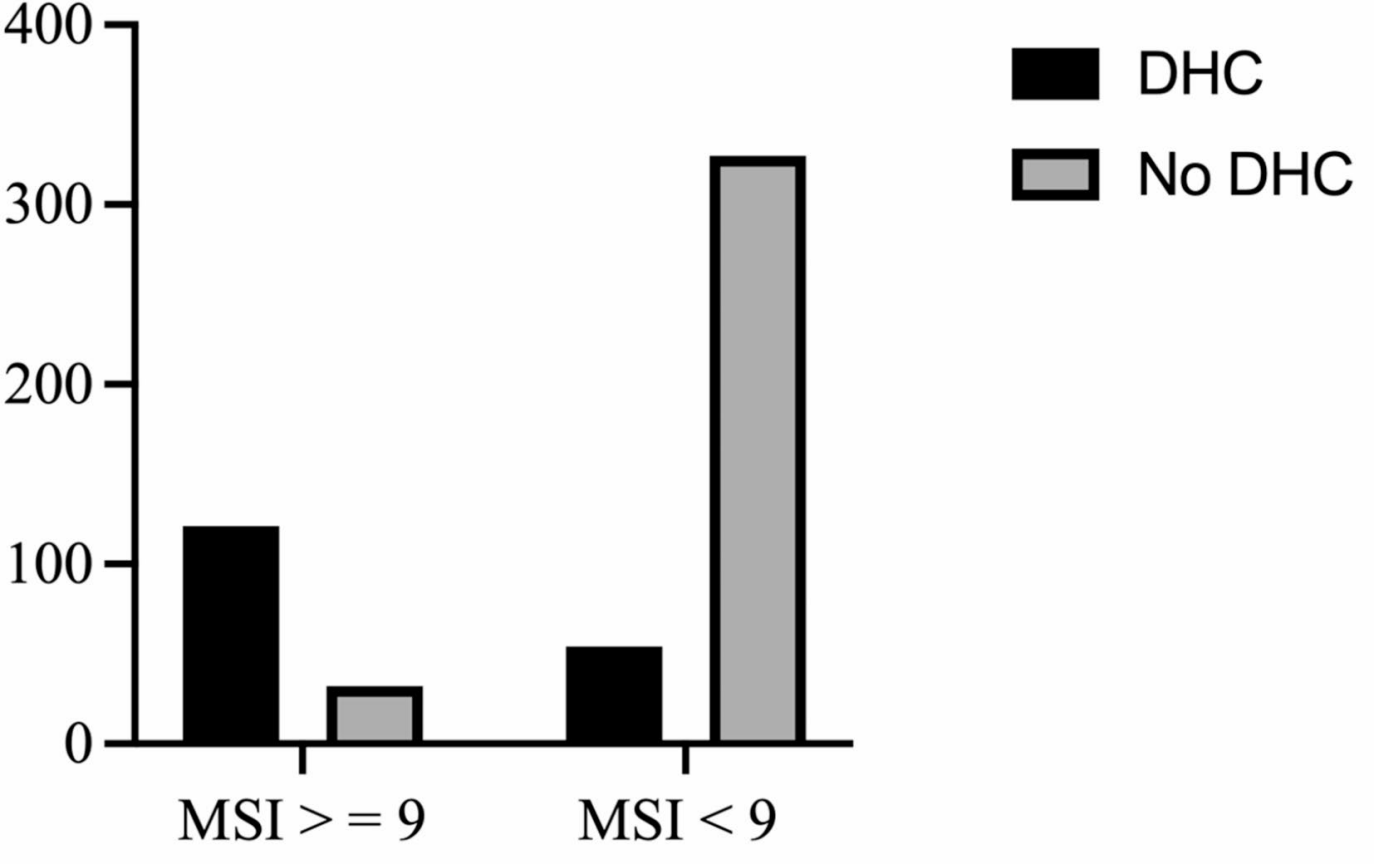
Patients with MSI-score of ≥ 9 had a 22-fold higher probability of requiring decompressive hemicraniectomy compared to those with MSI-score < 9 (Fisher’s Exact test, OR 22.90, 95%CI 14.08 to 37.48 sensitivity 69%, 95%CI 62–76%; specificity 91%, 95%CI 88–94%; positive predictive value 79%, 95%CI 72–85%; negative predictive value 86%, 95%CI 82–89%; *p* < 0.0001).

The following parameters were significantly associated with DHC after univariate analysis: poor collateral circulation (odds ratio 2.64, 95% 2.03 to 3.48, *p* = 0.008), no previous stroke (odds ratio 7.28, 95%CI 5.41 to 10.04, *p* = 0.03), age < 70 years (odds ratio 9.010, 95%CI 5.77 to 13.78, *p* < 0.0001), basal cistern effacement (odds ratio to infinity, 95%CI 18.26 to infinity, *p* < 0.0001), midline shift (odds ratio 33.95, 95%CI 16.28 to 69.38, *p* < 0.0001), no successful mechanical recanalization defined as TICI < 2b (odds ratio 10.50, 95%CI 6.77 to 16.31, *p* < 0.0001), NCCT ASPECTS < 7 (odds ratio 0.45, 95%CI 0.36 to 0.56, *p* < 0.0001), CBV ASPECTS < 6 (odds ratio 0.61, 95%CI 0.47 to 0.77, *p* < 0.0001), NIHSS > 16 on admission (odds ratio 0.77, 95%CI 0.62 to 0.94, *p* = 0.001). The results of the univariate analysis are summarized in Table 2. After performing the multivariate regression analysis, the following parameters were independent predictors: age < 70 years, basal cistern effacement, midline shift, no successful recanalization, and CBV ASPECTS < 6 (Table 3). A malignant stroke indicator (MSI) score ranging from 0 to 26 points was created based on these independent predictors. Points were assigned to each parameter according to their discrimination power (Table 3): For age < 70 years 7 points were assigned, for no successful recanalization 6 points were assigned, for basal cistern effacement 4 points were assigned, for CBV ASPECTS < 6, 3 points were assigned, for midline shift up to 6 points were assigned (Table 4). After performing ROC-analysis a MSI score with a cutoff value of 9 was found to have the highest discrimination power between patients needing DHC and those who don’t (AUC 0.90, 95%CI 0.87–0.93, *p* < 0.0001, Fig. 1). Patients with MSI-score ≥ 9 had a 22-fold higher probability of requiring DHC compared to those with MSI-score of < 9 (odds ratio 22.90, 95%CI 14.08 to 37.48, *p* < 0.0001, Fig. 2). The sensitivity was 0.69, 95%CI 0.62 to 0.75, the specificity was 0.91, 95%CI 0.88 to 0.94, the positive predictive value was 0.79, 95%CI 0.72 to 0.85, and the negative predictive value was 0.86, 95%CI 0.82 to 0.89.

**Table 2:** Univariate regression analysis.

Variables	Estimate	Standard error	95%CI	IZI	*p*-value
Poor collaterals (Souza et al. 0 to 2)	0.9593	0.3652	0.2834 to 1.729	2.627	0.008*
Age < 70 years	2.198	0.2232	1.770 to 2.647	9.851	< 0.0001*
Basal cistern effacement	0.7441	0.0932	0.5610 to 0.927	7.984	< 0.0001*
No previous stroke	0.7871	0.3779	0.0918 to 1.590	2.083	0.03*
No successful recanalization (TICI ≥ 2b)	2.358	0.2210	1.932 to 2.799	10.67	< 0.0001*
NCCT ASPECTS < 7 points	1.517	0.2064	1.118 to 1.928	7.349	< 0.0001*
CBV ASPECTS < 6 points	1.816	0.2994	1.249 to 2.429	6.063	< 0.0001*
NIHSS > 16 points	0.6424	0.1944	0.2635 to 1.026	3.304	0.0009*
Midline shift	3.525	0.3744	2.840 to 4.324	9.414	< 0.0001*

TICI = thrombolysis in cerebral infarction, NCCT = native cranial computed tomography, CBV = cerebral blood volume, ASPECTS = Alberta Stroke program early CT score, NIHSS = National Institutes of Health Stroke Scale; *significant results.

**Table 3:** Multivariate regression analysis model.

Variables	Estimate	Standard error	95% CI	ItI	*P* value
Poor collaterals (Souza et al. 0 to 2)	-0.01562	0.04165	-0.0975 to 0.0663	0.3750	0.7079
Age < 70 years	0.2344	0.03504	0.1654 to 0.3033	6.688	< 0.0001*
Basal cistern effacement	0.4042	0.09385	0.2196 to 0.5888	4.307	< 0.0001*
No previous stroke	0.06394	0.05509	-0.0444 to 0.1723	1.161	0.2466
No successful recanalization (TICI ≥ 2b)	0.2246	0.03838	0.1491 to 0.3001	5.852	< 0.0001*
NCCT ASPECTS < 7 points	0.02557	0.04151	-0.0560 to 0.1072	0.6160	0.5383
CBV ASPECTS < 6 points	0.1476	0.04298	0.0630 to 0.2322	3.435	0.0007*
NIHSS > 16 points	0.01180	0.03367	-0.0544 to 0.0780	0.3504	0.7262
Midline shift	0.08455	0.01375	0.0575 to 0.1116	6.147	< 0.0001*

TICI = thrombolysis in cerebral infarction, NCCT = native cranial computed tomography, CBV = cerebral blood volume, ASPECTS = Alberta Stroke program early CT score, NIHSS = National Institutes of Health Stroke Scale; *significant results.

**Table 4:** Malignant stroke Indicator (MSI-score).

Parameters	Presentation	Points
Age	≥ 70 years	0
	< 70 years	7
Basal cistern effacement	No	0
	Yes	4
Midline shift	0	0
	1–3 mm	1
	3–6 mm	2
	6–9 mm	4
	> 9 mm	6
Successful mechanical recanalization (TICI ≥ 2b)	No	0
	Yes	6
CBV ASPECTS	≥ 6	0
	< 6	3

TICI = thrombolysis in cerebral infarction, CBV = cerebral blood volume,

ASPECTS = Alberta stroke program early CT score.

### Subgroup analysis in the patient cohorts with successful vs. unsuccessful thrombectomy

In the patient cohort with a non-successful MT, the MSI score showed a high discrimination power (AUC 0.90, 95% CI 0.85–0.94, *p* < 0.0001, sensitivity 92%, specificity 79%). Patients with a MSI score cut-off of > 5.5 points had a significantly higher risk of requiring DHC (*p* < 0.0001, OR 0.085, 95% CI 0.01 to 0.06, PPV 0.87, NPV 0.85).

In the patient cohort with a successful MT, the MSI score showed a lower discrimination power (AUC 0.78, 95% CI 0.70–0.86, *p* < 0.0001, sensitivity 79%, specificity 72%). The optimal cut-off for the MSI score was > 10 points (*p* < 0.0001, OR 0.10, 95% CI 0.05 to 0.22, PPV 0.29, NPV 0.96).

### MSI score and functional outcome at 3 months follow up

Spearman correlation of mRS after 3 months with MSI score showed no significant correlation (*p* = 0.061). ROC analysis showed that the MSI score is partially able to discriminate between a good (mRS ≤ 3) and a bad outcome (mRS > 3) (AUC 0.5574, *p* = 0.0218, 95% CI 0.5086–0.6061, sensitivity 42.48%, specificity 69.78%). The cut-off value with the best discriminatory power was ≥ 10 points (*p* = 0.0067, OR 1.654, 95% CI 1.151 to 2.357). Patients with an MSI score of 10 points or more had a significantly lower risk of poor clinical outcome.

## Discussion

In this study, a comprehensive analysis of multiple potential predictors of malignant edema in stroke patients was performed and a scoring system (MSI score) for an early identification of patients at high risk for malignant stroke requiring DHC was elaborated. Regarding the need for DHC, the MSI score showed a high discriminatory power (AUC 0.90) with a cutoff value of 9 points in general and of 6 points in patients who did not receive successful MT. The MSI score was based on radiological and clinical parameters available on admission or at the latest after mechanical recanalization allowing a direct score calculation within 24 h after ictus, that can be considered for guidance of further treatment-decisions regarding the need for DHC.

Four independent risk factors are included in the MSI score, with age having the highest discriminatory power, followed by midline shift, success of the thrombectomy, effacement of the basal cisterns and the ASPECT score in CBV. Each parameter has a high discriminatory power, which has also been shown in previous studies. However, the informative value of the score is more robust than that of the individual parameters. None of the individual parameters alone like age or unsuccessful thrombectomy can justify the indication of perming DHC, while the presence of multiple parameters simultaneously indicating a high risk for developing malignant edema, can justify the indication for DHC. Since these are routinely recorded at an early stage anyway, the use of the MSI score should be preferred to an assessment of individual parameters when determining the indication for DHC. In contrast to the approach of previously published scoring systems to predict malignant edema formation in the setting of ischemic stroke, the MSI score presented here focusses on early identification of stroke patients requiring DHC, which is of great clinical importance to timely plan and initiate surgical interventions. Since such scoring system was lacking so far, a neurosurgical consultation concerning DHC in stroke patients happens at a late stage, when malignant edema already has manifested, and herniation is threatening to occur. In the following, we would like to compare the MSI score with other scores for the prediction of malignant brain edema. In summary, the MSI score has comparatively better discrimination power with a very good specificity of 91% but a relatively low sensitivity of 69%. Since our goal was to develop a score that can help determine an indication for surgery at an early stage, we considered a high specificity to be essential. Low sensitivity would be a problem primarily if the score were used to identify low-risk patients and consequently to monitor them less intensively. In this case, malignant edema requiring treatment could be missed. While the MSI score shares several features with the EDEMA score^9^, some parameters like the initial glucose level and the intravenous thrombolysis were no significant predictors of DHC in our study and were consequently excluded from the MSI score. The predictive power of the MSI score, with an AUC of 0.90, is higher than that of the EDEMA score of 0.76 (in a study population of 222 patients)^9^. Another score developed to predict malignant brain edema is the MBE score^14^. Mechanical thrombectomy, NIHSS and ASPECT are common features of the MBE score and the MSI score. The MBE score additionally considers the collateral score, which is calculated based on the leptomeningeal collateral circulation. The discrimination power of the MBE score for prediction of malignant edema was 0.88 (AUC) in a cohort of 121 patients. Interestingly, the collateral score was no independent predictor after performing a multivariate regression analysis. Although the NIHSS and the ASPECT score are commonly included parameters in several scoring systems, their discrimination power as a stand-alone score with AUC 0.74–0.78 was lower than that of MSI score^12^. The same applies for the NIHSS. In the study population analyzed for the MBE score, the AUC for the prediction of DHC was 0.75^14^. The MSI score includes MT, which has been a standard treatment for anterior circulation vascular occlusions since 2015 and thus plays an important role when it comes to outcome after ischemic stroke^15,16^. Aside from age, successful recanalization by conducting MT was the strongest discriminatory parameter regarding the need for DHC in our study population. Other prediction scores incorporate magnetic resonance imaging (MRI) as radiological modality^17,18^ - or computed tomography perfusion (CTP)^19^. Although diffusion-weighted imaging (DWI) is playing an increasing role in stroke diagnosis, MRI still has not been adopted as a standard imaging modality in stroke patients in most centers. For this reason, MRI was performed in only a very small number of stroke patients in our study, not allowing statistical analysis. The DASH score was developed based on MRI data, which considers the ASPECT score in DWI, as well as includes glucose level, a hyperemic media sign, and involvement of the anterior circulation territory^20^. In a study population of 119 patients, the AUC for the prediction of malignant brain edema was 0.88. The DASH score has not been evaluated regrading DHC so far. While most scoring system are mainly focusing on radiological signs of brain swelling, the Kasner Index also considers several comorbidities like arterial hypertension and heart failure, both of which have been identified as risk factors for malignant cerebral edema in ischemic stroke^21^. However, the patient number (201 patients) and the discriminatory power (AUC 0.70) of the Kasner Index study were lower compared to our study. The role of comorbidities as risk factors for malignant edema in stroke patients is controversially discussed in the literature. In a population of 114 patients, Dostovic et al. identified arterial hypertension, diabetes mellitus, and elevated creatinine as risk factors for the development of malignant edema^22^. On the other hand, one of the largest evaluations of the development of malignant cerebral edema in IS by Thorén et al. in a population of more than 42,000 patients showed that NIHSS, blood glucose level, hyperdense media sign, infarct sign at baseline, and reduced consciousness were the most important factors, whereas past medical history and blood pressure played a minor role^23^. Aside from the early identification of stroke patients at high risk for malignant edema requiring DHC it is of great importance to select patients, who will benefit from DHC regarding functional outcome. Patients with ischemic stroke developing malignant edema and receiving consequently DHC are expectedly those who also have worse outcome compared to the patients without malignant stroke. Therefore, not all parameters, that are predictors of malignant edema, are suitable to also identify the patients, who will benefit from DHC. In our study, the CBV ASPECTS providing an estimation of the volume of ischemic brain tissue was the only parameter that was significantly associated with functional outcome after DHC. The initial NIHSS also showed a trend to better outcome in patients with better clinical status on admission. ROC analysis showed that the MSI score is partially able to discriminate between a good (mRS ≤ 3) and a bad outcome (mRS > 3) (AUC 0.5574). Nonetheless, sensitivity is low at 42.48% and the MSI score was not primarily designed for the question of clinical outcome which is why the MSI score must not currently be used to make a statement about the extent of disability. A more reliable estimation of ischemic brain tissue with differentiation of ischemic core and penumbra based on CT perfusion or MRI (DWI) could contribute to a better risk stratification and selection of patients for DHC, who will benefit from this surgical procedure.

In summary, the MSI score is superior to other existing scores predicting the development of malignant edema in ischemic stroke, with an AUC of 0.90. In our opinion, it is therefore well suited to identify the patients needing DHC at an early stage. A limitation is the retrospective nature of the study since our indication for DHC is the basis for the development of the score. Furthermore, the score needs to be validated in an independent data set, which is to be conducted at a second center. Finally, a prospective study would be necessary in the next step to test the applicability of the score in a multicentric setting.

## Conclusion

The MSI score is a promising tool to predict the need for DHC at an early stage with the highest discriminatory power so far regarding DHC and merits a validation in an independent cohort and a prospective setting.

## Data Availability

Data included in this study can be requested from the corresponding author.

## Abbreviations

DHC: Decompressive craniectomy
IS: Ischemic stroke
FDCT: Non-enhanced flat panel detector computed tomography
FDCTA: Flat panel detector computed tomography angiography
MT: Mechanical thrombectomy
NCCT: Native cranial computed tomography
